# From Crisis to Complications: A Nationwide Cohort Study Assessing One-Year Cardiovascular and Thromboembolic Risks After Severe COVID-19 Compared to Matched Controls

**DOI:** 10.3390/jcm13237265

**Published:** 2024-11-29

**Authors:** Andreas Liliequist, Per Svensson, Robin Hofmann, Henrike Häbel, Marcus Ståhlberg, Per Nordberg

**Affiliations:** 1 Department of Medicine, Solna, Karolinska Institutet, 171 64 Stockholm, Sweden; marcus.stahlberg@ki.se; 2 Function Perioperative Medicine and Intensive Care, Karolinska University Hospital, 171 76 Stockholm, Sweden; per.nordberg@ki.se; 3 Department of Clinical Science and Education, Södersjukhuset, Karolinska Institutet, 118 83 Stockholm, Sweden; per.svensson@ki.se (P.S.); robin.hofmann@ki.se (R.H.); 4 Department of Learning, Informatics, Management and Ethics (LIME), Karolinska Institute, 171 65 Stockholm, Sweden; henrike.habel@ki.se; 5 Department of Physiology and Pharmacology, Karolinska Institute, 171 65 Stockholm, Sweden

**Keywords:** COVID-19, cardiovascular events, intensive care, long term outcome

## Abstract

**Background:** The long-term risk of cardiovascular and thrombotic events following severe COVID-19 remains largely unknown. This study aimed to assess the risk of atherosclerotic cardiovascular disease (ASCVD) within one year after hospital discharge in patients who received intensive care for severe COVID-19. **Methods:** A register-based nationwide case-control study on a cohort of patients with severe COVID-19 (cases) requiring mechanical ventilation and discharged alive without experiencing cardiovascular or thrombotic events during their hospital stay. Each case was matched (age, sex, district of residence) with up to 10 population-based controls. The primary outcome was ASCVD occurring after hospital discharge, defined as a composite endpoint, including myocardial infarction (MI), unstable angina pectoris and ischemic stroke. Secondary endpoints were MI, stroke, all-cause mortality, and venous thromboembolic events. Hazard ratio (HR) (95% CI) was used with adjustments for age, sex, socioeconomic factors, and co-morbidities. **Results:** In total, 31,375 individuals (70% men, median age 62 years) were included, of which 2854 had severe COVID-19 and 26,885 matched control subjects. The adjusted HR for ASCVD during the first year compared to control subjects was 3.1 (95% CI 1.7–5.4). Adjusted HRs for secondary outcomes for myocardial infarction were 2.0 (95% CI 0.8–5.3), for stroke 1.9 (95% CI 0.7–5.3), for pulmonary embolism 49.4 (95% CI 28.0–87.1), and deep venous thrombosis (DVT) 16.0 (95% CI 7.8–32.6). **Conclusions:** Severe COVID-19 requiring intensive care was associated with a substantial increase in 1-year risk for ASCVD and venous thromboembolic events.

## 1. Introduction

The novel SARS-CoV-2 virus has caused a global pandemic with almost 15 million excess deaths globally since the outbreak in 2020 [1]. Although most individuals experience mild flu-like symptoms, a substantial proportion of persons develop viral pneumonitis and acute respiratory distress syndrome with a need for supplemental oxygen and other respiratory support. About 10 percent of the patients admitted to hospital develop severe/critical COVID-19 requiring intensive care, often with the need for mechanical ventilation [2,3]. Severe COVID-19 is also associated with hyperinflammation characterized by fibroid necrosis and infiltration of lymphocytes and B-cells into cardiovascular wall cells, causing vasculitis in addition to endothelial dysfunction [4,5]. As more evidence emerged, treatment guidelines have been adjusted to respond to this inflammatory and hypercoagulable state [3]. These combined pathophysiologic mechanisms may increase cardiovascular risk after contracting COVID-19 [6,7]. In addition, risk factors (e.g., diabetes, male sex, etc.) for developing severe COVID-19 are shared for several cardiovascular diseases, both as comorbidities and complications [8].

Although some studies have reported increased 1-year cardiovascular risk following COVID-19, there is a need to assess the long-term risk for both atherosclerotic cardiovascular disease (ASCVD), thromboembolic events as well as other cardiac events (i.e., arrhythmias and heart failure) in this population to guide potential preventive measures [9,10,11,12,13,14,15,16,17]. The longer-term risk of incident cardiovascular disease after severe COVID-19 is not well characterized but is important to understand in order to recommend potential treatment and follow-up and put it into the context of actionable levels of ASCVD risk [18] and venous thromboembolic risk to inform treatment decisions on statins and oral anticoagulants. The primary aim of this study, using high-quality national registries, was to investigate whether severe COVID-19 requiring mechanical ventilation is independently associated with post-discharge risk of major cardiac events, including cardiovascular death, when controlling for several confounders, including socioeconomic factors.

## 2. Methods

### 2.1. Study Design

#### 2.1.1. Study Design and Ethics

We performed a nationwide cohort study based on data from the Swedish Intensive Care Registry (SIR) on patients (cases) with severe COVID-19 treated with mechanical ventilation and discharged alive from hospital between 1 March 2020 and 8 June 2021 [19,20,21,22]. For each case, 10 population-based controls were randomly selected from the Swedish Population Register and matched based on age, sex, and district of residence (defined as a subdivision of the municipality). The study database was then linked with various mandatory Swedish national registries maintained by Statistics Sweden and the National Board of Health and Welfare, using each individual’s unique personal identification number. The data used in the study were pre-collected and pseudonymized, ensuring minimal infringement on personal integrity. Ethical approval for the study was obtained from the Swedish Ethical Review Authority (Dnr 2020-01598). This study was conducted and reported in accordance with the STROBE (Strengthening the Reporting of Observational Studies in Epidemiology) guidelines to ensure transparency and rigor in observational research [23].

#### 2.1.2. National Registries and Data Collection

Eligible patients with severe COVID-19 were identified in the Swedish Intensive Care Registry (SIR), which covers approximately 95% of all ICU admissions in Sweden. Primary and secondary diagnoses from previous hospital admissions and outpatient visits, coded according to the International Classification of Diseases, 10th Revision (ICD-10), were retrieved from the National Patient Register (NPR). Socioeconomic and sociodemographic data, including income, education level, and country of birth, were obtained from the longitudinal integrated database for health insurance and labor market studies managed by Statistics Sweden.

#### 2.1.3. Inclusion Criteria

Consecutive patients who were diagnosed with severe COVID-19, defined as requiring mechanical ventilation (invasive or non-invasive) admission to a Swedish intensive care unit (ICU) and discharged alive from the hospital between 1 March 2020 and 8 June 2021, were included.

#### 2.1.4. Exclusion Criteria

Cardiovascular or Thrombotic Events During Hospitalization: Patients who experienced any cardiovascular or thrombotic events (such as myocardial infarction, stroke, deep vein thrombosis, or pulmonary embolism) during their hospital stay were excluded. This ensured that the study cohort represented individuals who were free from these outcomes at the time of hospital discharge. Mortality During Hospitalization: Patients who died during their hospital stay due to severe COVID-19 or related complications were excluded, as the study aims to assess long-term cardiovascular and thrombotic outcomes in survivors. Pre-existing Cardiovascular Disease: Individuals with a history of atherosclerotic cardiovascular disease (ASCVD), including myocardial infarction, stroke, or other major cardiovascular events, prior to hospitalization for COVID-19 were excluded to eliminate baseline confounding by pre-existing conditions. Any patients with missing or incomplete data regarding key variables (e.g., comorbidities, follow-up status) or those who were lost to follow-up within the first year post-discharge were excluded from the analysis. For control subjects, individuals who were registered in the Swedish Intensive Care Registry and had been admitted to the ICU with a diagnosis of COVID-19 were excluded.

#### 2.1.5. Control Group Selection

The control group consisted of individuals from the general population who were matched to the severe COVID-19 cases based on age, sex, and district of residence. These variables were chosen because they are strong demographic factors that can influence the risk of cardiovascular events and thromboembolic outcomes. By matching these characteristics, we aimed to create a control group that was similar to the COVID-19 cohort in terms of these potential confounders. For each severe COVID-19 case, 10 controls were selected who were within a range of the patient’s age, ensuring that the age distribution between the cases and controls was as similar as possible. The sex distribution of the controls was matched to the cases to control for gender differences in the incidence of cardiovascular and thrombotic events. The controls’ districts were also matched based on their district of residence, as this can account for regional differences in healthcare access, socioeconomic status, and local health behaviors, all of which can impact the risk of cardiovascular disease.

#### 2.1.6. Definition of Exposures

The primary exposure was severe COVID-19, which was defined as discharged alive from the hospital during the study period after at least one episode of treatment with mechanical ventilation in the ICU.

#### 2.1.7. Definition of Covariables and Variables for Subgroup Analyses

Conditions were identified based on diagnoses recorded in the National Patient Register (NPR) or prescriptions filled within the past 12 months. These conditions included hypertension (I10 or use of antihypertensive drugs), hyperlipidemia (E78 or use of lipid-lowering drugs), type 2 diabetes mellitus (E11 or use of antidiabetic drugs), type 1 diabetes mellitus (E10), obesity (E66), heart failure (I50.1, I50.9), atrial fibrillation (I48), venous thromboembolism (I26, I80), asthma (J45), chronic obstructive pulmonary disease (J44), chronic kidney disease (N18), malignancy (C, D40–48), rheumatoid arthritis (M05, M06), systemic inflammatory disease (M30–M36), and inflammatory bowel disease (K50, K51). A history of cardiovascular disease (CVD) was defined as a record of myocardial infarction (MI) (I21, I22), ischemic heart disease (I25), ischemic stroke (I63), or peripheral vascular disease (I70-I73) in the NPR (see Supplementary Table S1).

To adjust for socioeconomic status in the analyses, information on education level, region of birth, and disposable income was collected for all cases and control subjects. Education level was categorized as ≤9 years, 10–12 years, and >12 years based on the highest level attained in the year prior to admission. Region of birth was classified as within the European Union’s (EU) 15 countries (Austria, Belgium, Denmark, Finland, France, Germany, Greece, Ireland, Italy, Luxembourg, Netherlands, Portugal, Spain, Sweden, UK) and/or Nordic countries (Denmark, Finland, Iceland, Norway, Sweden) or outside these regions. Disposable income, reflecting average income per household consumption unit adjusted for household size and composition, was recorded for the year preceding admission. Income levels were categorized into quintiles specific to each calendar year, with the lowest quintile as the referent. Since median income was lower in women than in men, quintiles were also stratified by gender.

#### 2.1.8. Outcomes

The primary outcome was a composite of ASCVD occurring after hospital discharge as adopted from the New Pooled Cohort Risk Equations [24], defined as a fatal or non-fatal myocardial infarction, unstable angina pectoris requiring urgent revascularization, fatal and non-fatal ischemic stroke, and cardiovascular death registered in the National Patient Register or the Cause of Death Register during the follow-up period. The ICD-10 coding used to classify the outcome is provided in Supplementary Table S1. Secondary endpoints were pre-specified as fatal or non-fatal MI, fatal or non-fatal stroke, all-cause mortality, venous thromboembolic events (PE and DVT), pericarditis, and myocarditis. The last date of follow-up was 15 August 2021.

### 2.2. Statistical Methods

Categorical variables are reported as frequencies and percentages, while continuous variables are reported as median and interquartile range (IQR). Hazard ratios (HR) and 95% confidence intervals (CI) for the association between the exposure and the different outcomes were calculated by means of the Cox regression, both crude and adjusted for age (in splines), sex, level of education, marital status, income quintile, and comorbidities; hypertension, hyperlipidemia, diabetes 1 and 2, obesity, chronic kidney disease, chronic obstructive pulmonary disease (COPD), asthma and malignancy. Statistical analyses were performed using Stata version 16.1 (StataCorp, College Station, TX, USA).

## 3. Results

**Figure 1:**
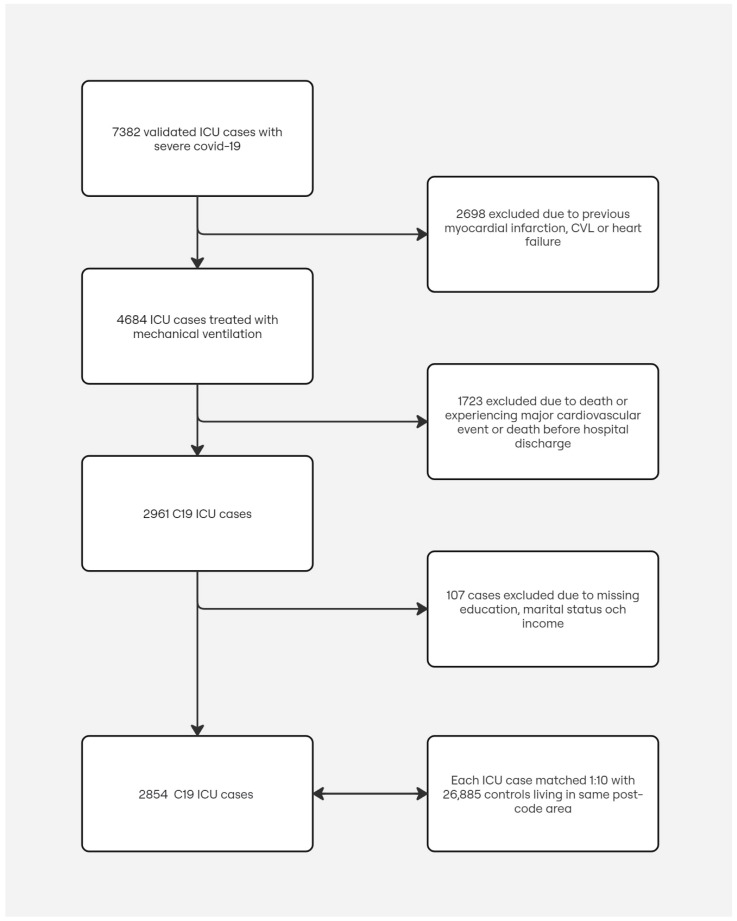
Patient flowchart.

During the study period between 1 March 2020 and 8 June 2021, we included 2854 patients with severe COVID-19 treated with mechanical ventilation at a Swedish ICU, discharged alive from the hospital without experiencing any cardiovascular or thrombotic events during the hospitalization period. For each patient, up to 10 control subjects matched for age, sex, and district or residence were randomly selected, rendering a total of 26,885 control subjects. The study population selection procedure and reasons for exclusions are shown in Figure 1. Baseline characteristics for cases and the matched control subjects are summarized in Table 1.

### 3.1. Cardiovascular Events

During the first 12 months of follow-up, the incidence rate of ASCVD per 1000 patient-years was 9.9 (6.2–15.7) in patients discharged after severe COVID-19 compared to 2.6 (2.0–3.5) in matched control subjects (Table 2). Thus, the risk for incident ASCVD during follow-up was increased compared to control subjects, HR (95% CI), 3.7 (2.2–6.4). When adjusting for important confounders such as cardiovascular risk factors and comorbidities, enriched among cases, as well as socioeconomic factors, the increased risk compared to matched control subjects was similar 3.1 (1.7–5.4). The multivariable-adjusted HRs with 95% CIs for incident myocardial infarction and stroke were 2.0 (0.8–5.3) and 1.9 (0.7–5.0), respectively.

### 3.2. Thromboembolic Events

During follow-up, the incidence rate of pulmonary embolism per 1000 patient-years was 47.0 (28.0–87.1) in patients discharged after severe COVID-19 compared to 0.9 (0.5–1.5) in matched control subjects. The corresponding incidence rates of deep venous thrombosis discharged after severe COVID-19 and controls were 14.1 (9.9–20.2) and 0.7 (0.6–1.4), respectively. Thus, the risk for incident thromboembolic events during the first year was clearly increased compared to control subjects; the multivariable-adjusted HRs with 95% CIs for pulmonary embolism and DVT were 49.4 (28.0–87.1) and 16.0 (7.8–32.6), respectively.

### 3.3. Perimyocarditis

During follow-up, the incidence rate of myocarditis per 1000 patient-years was 1.1 (0.3–4400.0) in patients discharged alive after severe COVID-19 compared to 0.1 (0.0–0.4) in matched control subjects. The corresponding incidence rates of pericarditis discharged after severe COVID-19 and controls were 1.6 (0.5–5.1) and 0.4 (0.2–0.8), respectively. Thus, the risk for incident perimyocarditis during the first year may increase compared to control subjects; the multivariable-adjusted HRs with 95% CIs for myocarditis and pericarditis were 33.9 (1.1–1089.5) and 3.6 (0.9–14.8), respectively.

### 3.4. Mortality

**Figure 2:**
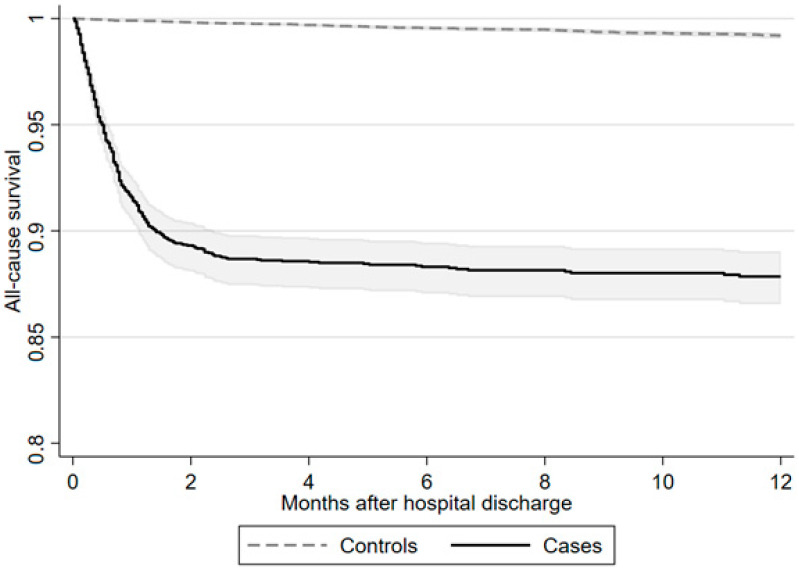
All-cause survival after hospital discharge.

During follow-up, the incidence rate of mortality per 1000 patient-years was clearly increased compared to control subjects, with 184.9 (166.3–205.7) after severe COVID-19 compared to 8.4 (7.2–9.8) in matched control subjects. Explorative 1-year all-cause mortality is presented in Supplementary Materials as a Kaplan–Meier curve (Figure 2).

## 4. Discussion

This nationwide cohort study using matched control found that patients with severe COVID-19 requiring mechanical ventilation in the ICU were associated with an increased risk for adverse cardiovascular events, including atherosclerotic cardiovascular disease (ASCVD) such as myocardial infarction and stroke, as well as venous thromboembolism within one year after hospital discharge. These findings may have implications for clinical practice and public health.

The elevated risk for ASCVD observed in ICU patients may be influenced not only by the prolonged inflammatory response associated with COVID-19 but could also reflect a broader risk associated with critical illness or treatments administrated during their hospital stay. There is substantial literature documenting long-term cardiovascular complications following sepsis, both bacterial [25] and viral [26], which share similar pathophysiological pathways with severe COVID-19. The systemic inflammation, immune dysregulation, and endothelial injury characteristic of sepsis are also prominent features of severe COVID-19, suggesting a possible overlap in the mechanisms driving long-term cardiovascular risk in these populations. Additionally, the Renin–Angiotensin–Aldosterone System (RAAS) and Kinin–Kallikrein System have been implicated in the pathophysiology of COVID-19 and could contribute to the observed cardiovascular complications [27]. These systems, which play a role in blood pressure regulation, fluid balance, and inflammatory responses, may be dysregulated in severe COVID-19, leading to an increased risk of cardiovascular events.

In our study, we have not been able to adjust for a potential beneficial effect or risk of certain treatments on cardiovascular events. For instance, statin therapy prior to hospital admission in COVID-19 patients has been associated with lower levels of systemic inflammation and acute kidney injury [28,29]. Also, corticosteroids, while essential in managing severe COVID-19, have well-documented effects on cardiovascular risk, including promoting hyperglycemia, dyslipidemia, and hypertension. Although our study did not adjust for these treatments, it is crucial to consider their potential contribution to the increased cardiovascular risk.

The differentiation between the various types of cardiovascular events is probably important for tailoring follow-up and preventive strategies in this high-risk population. While the associated risk for both ASCVD and thromboembolic events was significantly higher in ICU patients, the degree of risk and the temporal dynamics may differ between these events. For instance, several studies have highlighted that the risk for thromboembolic events, particularly venous thromboembolism (VTE), which is acutely elevated during the active phase of COVID-19, particularly during hospitalization. This association, although diminished over time, remains elevated up to a year after discharge. In contrast, the risk for myocardial infarction and stroke may persist due to chronic inflammatory responses and potential endothelial dysfunction post-infection [30].

The risk association for thromboembolic events in non-severe COVID-19 is comparable with pre-pandemic observations showing strong associations between recent respiratory infection and myocardial infarction or thromboembolism [31]. Thus, an unregulated immune response in the critically ill may result in an increased risk for venous thrombosis [32,33,34].

There is limited knowledge of how the risk association of cardiovascular events differs between COVID-19 patients and the general ICU population. Larsson et al. showed that the risk profiles for acquiring severe COVID-19 and ARDS due to Influenza A are similar [8]. However, less is known about the long-term effects of cardiovascular risk [6]. Sepsis requiring critical care is increasingly recognized as a risk factor for subsequent cardiovascular events, with about one-third of sepsis survivors hospitalized for cardiovascular complications within a year of their illness. Thus, this could suggest that our finding may be a consequence of critical illness rather than COVID-19 per se. Increased systemic inflammation may explain the link between sepsis and cardiovascular events, but the exact mechanisms are still largely unknown.

The last follow-up date was set for 15 August 2021. This date was chosen primarily to allow for a sufficient follow-up period of up to one year post-discharge for patients included in the earlier phases of the pandemic, which in Sweden included most patients with severe COVID-19. However, this choice of date introduces some complexities, particularly in the context of the evolving pandemic, new treatments, the emergence of new COVID-19 variants, and the rollout of vaccinations. Vaccination against COVID-19 began in late 2020, and by mid-2021, a large proportion of the population had received at least one dose. Vaccination has been shown to reduce not only the risk of new COVID-19 infections but also to reduce the likelihood of severe complications, including cardiovascular events associated with COVID-19 [35]. As a result, our cohort likely includes both vaccinated and unvaccinated patients, which may introduce variability in the observed outcomes. While this study did not specifically stratify patients by vaccination status, future research should aim to account for this variable to assess the impact of vaccination more accurately on long-term cardiovascular outcomes post-COVID-19.

## 5. Limitations

First, the cohort only includes patients treated in an intensive care setting with mechanical ventilation, excluding other hospitalized patients with severe COVID-19 who did not require ICU care. This may affect the generalizability of our findings to all hospitalized COVID-19 patients.

Second, our analysis did not adjust for the potential impact of treatments like corticosteroids, ACE inhibitors, statins, or vaccines on cardiovascular outcomes post-discharge, which may significantly confound the observed association between severe COVID-19 and increased cardiovascular risk.

Third, the study relies on data from the Swedish national registry, which did not include specific information on COVID-19 status for the control group, potentially leading to misclassification if some control patients had undiagnosed COVID-19.

Fourth, our study lacks a comparison group of patients with other types of severe pneumonia requiring mechanical ventilation, limiting our ability to distinguish the specific effect of COVID-19 from those in critical illness in general. Additionally, the matching process, while necessary to create comparable groups, introduces constraints on the extrapolation of our findings to the broader population.

Sixth, big database studies like ours inherently carry limitations, such as potential biases in data entry, missing data, and the ability to control for all confounding variables.

Moreover, while the study provides valuable insights into the long-term risks of cardiovascular and thrombotic events following severe COVID-19, the generalizability of the results may be limited by the following factors: The study predominantly focuses on middle-aged, severely ill COVID-19 patients, which may not be fully representative of the broader population or patients with mild disease. The intensive care context and post-discharge care practices in Sweden may differ from other countries, potentially limiting the applicability of the findings in different settings. The relatively short follow-up period (one year) may not capture long-term risks, and changes in COVID-19 variants could alter the generalizability of these results in the future.

## 6. Conclusions

In this nationwide cohort study, severe COVID-19 requiring intensive care was associated with a substantial increase in 1-year risk for specific ASCVD and venous thromboembolic events compared to the general population. These findings highlight the need for close monitoring and potential preventive measures in patients discharged after severe COVID-19, with a focus on managing and mitigating the risk of these complications.

## Figures and Tables

**Table 1:** Baseline characteristics for cases and matched control subjects.

	Controls	Cases	Total	*p*-Value
	N = 26,885	N = 2854	N = 29,739	
Age	60.00 (52.00–69.00)	61.00 (53.00–69.00)	61.00 (52.00–69.00)	<0.001
Age categorized				0.003
Young	17,002 (63.2%)	1723 (60.4%)	18,725 (63.0%)	
Old	9883 (36.8%)	1131 (39.6%)	11,014 (37.0%)	
Sex				0.12
Male	18,436 (68.6%)	1998 (70.0%)	20,434 (68.7%)	
Female	8449 (31.4%)	856 (30.0%)	9305 (31.3%)	
Education				<0.001
≤9 yrs	5060 (18.8%)	795 (27.9%)	5855 (19.7%)	
9–12 yrs	12,393 (46.1%)	1290 (45.2%)	13,683 (46.0%)	
≥12 yrs	9432 (35.1%)	769 (26.9%)	10,201 (34.3%)	
Civil status				<0.001
Unmarried	13,028 (48.5%)	1268 (44.4%)	14,296 (48.1%)	
Married	13,857 (51.5%)	1586 (55.6%)	15,443 (51.9%)	
Level of income				<0.001
Quintile 1	5007 (18.6%)	777 (27.2%)	5784 (19.4%)	
Quintile 2	4729 (17.6%)	576 (20.2%)	5305 (17.8%)	
Quintile 3	5559 (20.7%)	553 (19.4%)	6112 (20.6%)	
Quintile 4	5865 (21.8%)	501 (17.6%)	6366 (21.4%)	
Quintile 5	5725 (21.3%)	447 (15.7%)	6172 (20.8%)	
EU-origin (EU-15)				<0.001
Non EU-15	5342 (19.9%)	981 (34.4%)	6323 (21.3%)	
EU-15	21,542 (80.1%)	1868 (65.6%)	23,410 (78.7%)	
Duration of hospitalization		8.00 (2.00–15.00)	8.00 (2.00–15.00)	
Hypertension	9139 (34.0%)	1405 (49.2%)	10,544 (35.5%)	<0.001
Hyperlipedemia	4094 (15.2%)	586 (20.5%)	4680 (15.7%)	<0.001
Diabetes 2	2651 (9.9%)	675 (23.7%)	3326 (11.2%)	<0.001
Diabetes 1	107 (0.4%)	12 (0.4%)	119 (0.4%)	0.86
Obesity	848 (3.2%)	472 (16.5%)	1320 (4.4%)	<0.001
Chronic Kidney disease	228 (0.8%)	92 (3.2%)	320 (1.1%)	<0.001
Atrial fibrillation	936 (3.5%)	195 (6.8%)	1131 (3.8%)	<0.001
COPD	411 (1.5%)	98 (3.4%)	509 (1.7%)	<0.001
Asthma	2745 (10.2%)	577 (20.2%)	3322 (11.2%)	<0.001
Malignancy	4142 (15.4%)	482 (16.9%)	4624 (15.5%)	0.038
Time to death	130.50 (53.00–257.00)	19.00 (9.00–34.00)	27.00 (12.00–88.00)	<0.001
Time to event	256.00 (159.00–475.00)	239.00 (134.00–468.00)	254.00 (157.00–474.00)	<0.001
Death (anytime)	174 (0.6%)	343 (12.0%)	517 (1.7%)	<0.001
Death within 30 days	27 (0.1%)	240 (8.4%)	267 (0.9%)	<0.001
Death within 90 days	63 (0.2%)	323 (11.3%)	386 (1.3%)	<0.001
Death within 180 days	109 (0.4%)	332 (11.6%)	441 (1.5%)	<0.001
Death within 1 year	155 (0.6%)	339 (11.9%)	494 (1.7%)	<0.001

Data are presented as median (IQR) for continuous measures, and n (%) for categorical measures.

**Table 2:** Incidence rates and hazard ratios for cardiovascular events during the first 12 months after severe COVID-19.

Outcomes		Events	Person Years	Incidence Rate per 1000 Person-Years	Crude HR (95% CI)	Multivariable Model ^†^ HR (95% CI)
ASCVD	Control	50	18,984	2.6 (2.0–3.5)	1.00	1.00
	Cases	18	1822	9.9 (6.2–15.7)	3.7 (2.2–6.4)	3.1 (1.7–5.4)
Myocardial infarction	Control	27	19,001	1.4 (1.0–2.1)	1.00	1.00
	Cases	6	1829	3.3 (1.5–7.3)	2.3 (1.0–5.6)	2.0 (0.8–5.3)
Stroke	Control	23	19,003	1.2 (0.8–1.8)	1.00	1.00
	Cases	5	1830	2.7 (1.1–6.6)	2.3 (0.9–6.0)	1.9 (0.7–5.0)
Myocarditis	Control	1	19,010	0.1 (0.0–0.4)	1.00	1.00
	Cases	2	1831	1.1 (0.3–4400.0)	19.7 (1.9–207.7)	33.9 (1.1–1089.5)
Pericarditis	Control	7	18,994	0.4 (0.2–0.8)	1.00	1.00
	Cases	3	1830	1.6 (0.5–5.1)	4.4 (1.1–17.0)	3.6 (0.9–14.8)
Pulmonary embolism	Control	16	17,997	0.9 (0.5–1.5)	1.00	1.00
	Cases	79	1682	47.0 (37.7–58.6)	50.1 (29.4–85.4)	49.4 (28.0–87.1)
DVT	Control	15	18,732	0.8 (0.5–1.3)	1.00	1.00
	Cases	24	1789	13.2 (9.0–20.0)	16.2 (8.6–30.1)	16.0 (7.8–32.6)

HR = hazard ratio. CI = Confidence Interval. ^†^ = adjusted for age, gender, all comorbidities reported in Table 1, level of education, marital status, and income quintile.

## Data Availability

Data cannot be made available due to legal reasons in Sweden.

## Supplementary Materials

The following supporting information can be downloaded at: https://www.mdpi.com/article/10.3390/jcm13237265/s1. Tables S1 and S2: Outcome definitions. Table S3: Logistic regression with presentation of the single independent variables Age, gender, comorbidities and socioeconomic status and association with ASCVD. Table S4: Characteristics by ASCVD 1 yr (cases with no event vs. cases with event).
