# Supplementary material for: From Crisis to Complications: A Nationwide Cohort Study Assessing One-Year Cardiovascular and Thromboembolic Risks After Severe COVID-19 Compared to Matched Controls
Source: J Clin Med. 2024 Nov 29;13(23):7265. doi: 10.3390/jcm13237265 (PMC11641933; doi:10.3390/jcm13237265)
Supplement: Supplementary file 1 [file jcm-13-07265-s001.zip › jcm-3284922-supplementary.pdf]

Supplemental Tables

Table S1 – Outcome definitions

<table>
<tr><th colspan="3">Outcomes</th><th>ICD-10 code</th><th>Registry</th></tr>
<tr><td rowspan="5">ASCVD</td><td colspan="2">Nonfatal MI</td><td>I210-I214 & I219, I220-221, I228-I229</td><td>Inpatient</td></tr>
<tr><td rowspan="2">CHD death</td><td>Fatal MI</td><td>I210-I214 & I219, I220-221, I228-I229</td><td>Cause of Death</td></tr>
<tr><td>Sudden cardiac death</td><td>I461, I469</td><td>Cause of Death</td></tr>
<tr><td colspan="2">Fatal ischemic stroke</td><td>I630-I635, I638-I639</td><td>Cause of Death</td></tr>
<tr><td colspan="2">Nonfatal ischemic stroke</td><td>I630-I635, I638-I639</td><td>Inpatient</td></tr>
</table>

Abbreviations: ICD-10, international classification of diseases, 10th edition; ASCVD, atherosclerotic cardiovascular disease; MI, myocardial infarction; CHD, coronary heart disease.

Table S2 Secondary outcomes

Outcomes	ICD-10 code	Registry
Nonfatal MI	I210-I214 & I219, I220-221, I228-I229	Inpatient
Nonfatal PE	I260-I269	Inpatient
Nonfatal DVT	I800-I809	Inpatient
Nonfatal ischemic stroke	I630-I649	Inpatient
All-cause death	All	Cause of Death

Abbreviations: ICD-10, international classification of diseases, 10th edition; ASCVD, atherosclerotic cardiovascular disease; MI, myocardial infarction; CHD, coronary heart disease.

Table S3. Logistic regression with presentation of the single independent variables Age, gender, comorbidities and socioeconomic status and association with ASCVD.

Outcome	Odds Ratio	CI 95%	P-value
Cases	**2.6**	**(1.5-4.7)**	**0.001**
Age	1.1	(1.0-1.1)	0.001
Sex	1.0	(1.0-1.1)	0.882
Hypertension	1.3	(0.7-2.4)	0.442
Hyperlipedemia	1.0	(0.5-1.9)	0.974
Diabetes type 2	2.2	(1.2-3.9)	0.012
Obesity	1.3	(0.6-3.1)	0.486
Chronic kidney disese	1.8	(0.6-5.6)	0.346
Atrial fibrillation	1.3	(0.6-3.0)	0.496
COPD	2.4	(1.0-6.1)	0.064
Asthma	0.9	(0.4-1.7)	0.677
Malignancy	1.1	(0.6-2.0)	0.718
Education			
9-12 yrs	1.5	(0.8-2.8)	0.250
>=12 yrs	1.6	(0.8-3.2)	0.208
Civil status			
Married	0.8	(0.5-1.3)	0.364
Level of income			
Quintile 2	1.1	(0.5-2.2)	0.819
Quintile 3	0.7	(0.3-1.6)	0.378
Quintile 4	1.0	(0.5-2.1)	0.934
Quintile 5	0.7	(0.3-1.6)	0.437

Table S4. Characteristics by ASCVD 1yr (cases with no event vs cases with event)

	0<br>N=2,789	1<br>N=65	Total<br>N=2,854	p-value
Age	61.00 (52.00-69.00)	63.00 (56.00-71.00)	61.00 (53.00-69.00)	0.19
Age category				0.57
young	1,686 (60.5%)	37 (56.9%)	1,723 (60.4%)	
old	1,103 (39.5%)	28 (43.1%)	1,131 (39.6%)	
Gender				0.009
male	1,943 (69.7%)	55 (84.6%)	1,998 (70.0%)	
female	846 (30.3%)	10 (15.4%)	856 (30.0%)	
Level of education				0.63
<=9 yrs	780 (28.0%)	15 (23.1%)	795 (27.9%)	
9-12 yrs	1,260 (45.2%)	30 (46.2%)	1,290 (45.2%)	
>=12 yrs	749 (26.9%)	20 (30.8%)	769 (26.9%)	
Civil status				0.98
Unmarried	1,239 (44.4%)	29 (44.6%)	1,268 (44.4%)	
Married	1,550 (55.6%)	36 (55.4%)	1,586 (55.6%)	
Level of income				0.11
Quintile 1	764 (27.4%)	13 (20.0%)	777 (27.2%)	
Quintile 2	567 (20.3%)	9 (13.8%)	576 (20.2%)	
Quintile 3	540 (19.4%)	13 (20.0%)	553 (19.4%)	
Quintile 4	488 (17.5%)	13 (20.0%)	501 (17.6%)	
Quintile 5	430 (15.4%)	17 (26.2%)	447 (15.7%)	
EU origin (EU15)				0.53
Non EU15	961 (34.5%)	20 (30.8%)	981 (34.4%)	
EU15	1,823 (65.5%)	45 (69.2%)	1,868 (65.6%)	
Duration of hospitalization	8.00 (2.00-15.00)	6.00 (2.00-14.00)	8.00 (2.00-15.00)	0.52
Hypertension	1,369 (49.1%)	36 (55.4%)	1,405 (49.2%)	0.32
Hyperlipidemia	570 (20.4%)	16 (24.6%)	586 (20.5%)	0.41
Diabetes 2	659 (23.6%)	16 (24.6%)	675 (23.7%)	0.85
Diabetes 1	12 (0.4%)	0 (0.0%)	12 (0.4%)	0.60
Obesity	461 (16.5%)	11 (16.9%)	472 (16.5%)	0.93
Chronic Kidney disease	89 (3.2%)	3 (4.6%)	92 (3.2%)	0.52
Atrial fibrillation	187 (6.7%)	8 (12.3%)	195 (6.8%)	0.077
COPD	96 (3.4%)	2 (3.1%)	98 (3.4%)	0.87
astma_all	568 (20.4%)	9 (13.8%)	577 (20.2%)	0.20
Malignancy	471 (16.9%)	11 (16.9%)	482 (16.9%)	0.99
Time to death	19.00 (9.00-34.00)	23.00 (11.00-73.00)	19.00 (9.00-34.00)	0.39
Time to event	237.00 (134.00-466.00)	434.00 (228.00-486.00)	239.00 (134.00-468.00)	0.004
Death (anytime)	330 (11.8%)	13 (20.0%)	343 (12.0%)	0.045
Death within 30 days	232 (8.3%)	8 (12.3%)	240 (8.4%)	0.25
Death within 90 days	313 (11.2%)	10 (15.4%)	323 (11.3%)	0.30
Death within 180 days	320 (11.5%)	12 (18.5%)	332 (11.6%)	0.082
Death within 1 year	326 (11.7%)	13 (20.0%)	339 (11.9%)	0.041

Data are presented as median (IQR) for continuous measures, and n (%) for categorical measures.
